# How Talent Acceleration Generated Talent Advocacy

**DOI:** 10.1097/HS9.0000000000000046

**Published:** 2018-05-18

**Authors:** Deepa Maas-Sundararaman, Esther Sifuma

**Affiliations:** European Hematology Association, Executive Office, The Hague, The Netherlands

**Figure:**
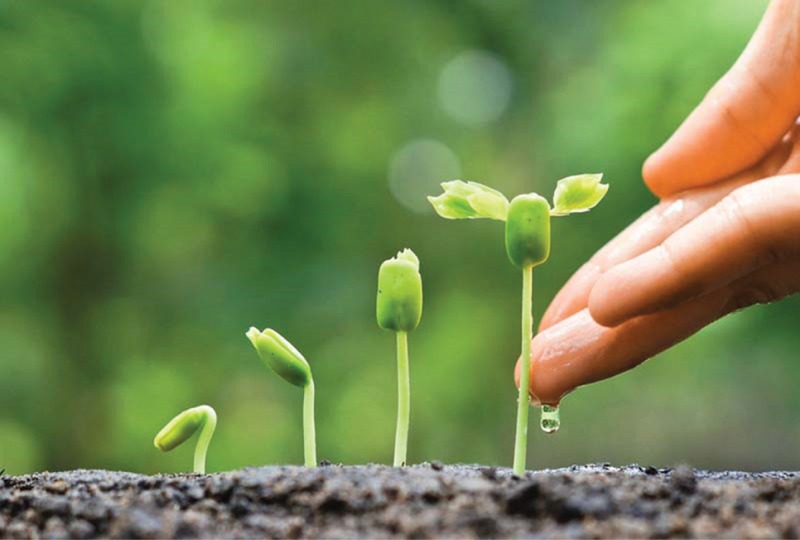


**How Talent Acceleration Generated Talent Advocacy.**

“Truly career changing. If I had to choose, I would choose mentorship over funding. It has been of such value to my career.”

Hearing this from a young researcher makes one wonder what type of mentoring would be so impactful to justify such a bold claim.

The answer can be found at the Spring Course of the EHA-ASH Translational Research Training in Hematology (TRTH).

Where adequate leaders show how it is done by listing their successes, the faculty at TRTH are top-tier experts of a special phenotype: excellent leaders who share the struggles they coped with and how they managed or learned from failures. This willingness to be vulnerable sets the scene for an atmosphere of trust and fosters an understanding amongst the talents that the venerated faculty members were once just like them. The line between the 20 talents that makes it into the program and the 13+ faculty members are soon blurred. This group of peers challenges each other's thinking, career choices and surprisingly, give feedback that only now the other is willing to receive.

The research projects that the talents present are scrutinized from hypothesis, choice of endpoints, technique, choice of models, statistics, control, and funding to ethics. This can be rather overwhelming for some. Fortunately, most have the strength of character to straighten their back and work on whatever they need to work on to achieve a new level of excellence, sparring with whichever faculty member or fellow talent with the necessary expertise. The daytime didactic sessions are known to elevate the knowledge of the talents science-wise. The evening talks detail the faculty's unembellished career paths, making the participants relate to these role models.

The talents leave the Spring Course inspired and with a completely reworked research project. It is not surprising that most remain in touch with and continue to collaborate with their mentors and peers for years after they participated in TRTH.

Currently, the call is open for the 10th edition of TRTH.

EHA received many requests to run a similar program in clinical research and in 2016, EHA's Clinical Research Training in Hematology (CRTH) was born. CRTH closely mirrors the mentoring aspect of TRTH. Every year, 15 talented clinical researchers are accepted into the program. Around 13 top-tier experts in various aspects of clinical research serve as faculty. The faculty mentors the talents to achieve excellence through a holistic mastery of the clinical trial ecosystem. To accomplish this, 2 strategies are employed.

The first is by discussing gray areas in clinical research. Knowledge of the basics can lead to adequate performance. Regulation and protocols as well as habits have created a landscape of black and white. However, there are areas that are not clearly defined: the gray areas. The experiences of faculty provide the tools for smart navigation of the gray areas, leading to reduction of waste and achievement of excellence.

The second strategy is to widen the scope to include other critical aspects that may have been neglected because they are not hard science: how to make a budget, negotiate funding, and how to engage collaborators.

CRTH participants discover at the first workshop what they did not know.

The participants bring their raw projects to the second workshop and leave this workshop with a refined project, including a realistic budget and a negotiation plan. They leave having made lifelong connections with their peers and faculty, connections that will likely impact their careers positively for years to come. More importantly, they leave with the magnanimous realization that they need a team to get it done, that they cannot and should not do this alone.

Currently, the call is open for the 3rd year of CRTH.

“Upon my return from the USA, the EHA Research Grant helped me start my own research group.”

Besides mentoring, funding is a crucial aspect in facilitating quality research in Europe. EHA funds talented researchers with grants, ranging from €100,000 to €160,000.

These research grants help talented postdocs to not only boost their careers but also often to achieve independence as a researcher. The flexibility of these grants address a need in the competitive environment of research funding for young researchers.

It is gratifying to note that 1 of the Editors-in-Chief of this journal, Jan Cools, received an EHA Research Grant in 2004 and is 1 of almost 100 recipients of EHA funding since 2000.

The call for the research grants will open in fall.

The value of mobility and collaboration to a researcher's career is undisputed. Cognizant of this value, EHA, is keenly invested in facilitating its young members to experience different ways of working and learn new techniques.

EHA makes this investment through the Research Mobility Grants. These awards are geared toward a younger demographic. EHA will facilitate short-term visits to a research group in another institute that has complementary expertise. These visits promote collaboration between the institutes. PhD students up to postdocs can apply.

The call for Research Mobility Grants is open 3 times a year.

To foster collaboration between European and Japanese institutes, EHA together with the Japanese Society for Hematology (JSH) facilitates supplies, travel and accommodation for valuable scientific exchanges, and welcomes applications from young researchers with interesting research ideas.

The call for the EHA-JSH Fellowship Exchange Program is open permanently.

More information and eligibility criteria of EHA Talent Accelerator programs can be found via www.ehaweb.org/YoungEHA.

## Talent advocacy

A group of TRTH Alumni and past grant winners met and shared their stories of how winning an EHA Award had impacted their careers.

Wanting to give back, they formed a group and offered to advise EHA on matters relevant to young hematologists and researchers. They initially proposed to offer advice to the EHA Talent Accelerator (previously known as Career Development), and this quickly but organically expanded to other EHA activities such as the Congress of EHA and educational initiatives after the realization that indeed young EHA issues are part of every aspect of EHA.

With this enthusiasm for representing the next generation, the EHA Board asked the group to formally become an EHA committee.

## YoungEHA committee

**Figure:**
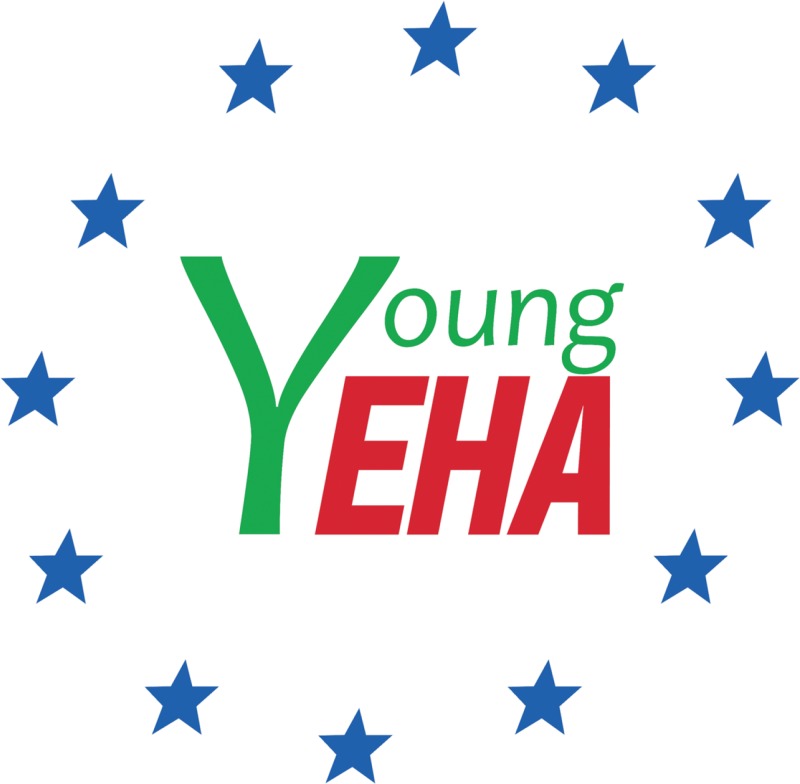


**The YoungEHA logo identifies EHA activities organized for early career researchers and physicians.**

V. Gaidzik, Germany (Chair); H. Becker, Germany; A.-K. Eisfeld, USA; M. Gruber, Austria; F. McClanahan, USA; M. Mraz, Czech Republic; C. Scharenberg, Sweden.

If you are a young researcher, a young clinician, or otherwise involved in hematology in the early phase of your career, consider yourself part of the YoungEHA community, which the YoungEHA Committee represents.

You will get to meet the YoungEHA Committee in a future issue of HemaSphere and when attending the inspiring YoungEHA track at the 23rd Congress of EHA in Stockholm, June 14–17, 2018.

